# The hidden hazards of clay: a qualitative exploration of silica exposure and well-being in ceramic art studios

**DOI:** 10.1080/17482631.2026.2661147

**Published:** 2026-04-17

**Authors:** Charles Vicku, Albert Essuman, Samuel Nii Adamah Sampah, Kofi Adjei, Samuel Nortey, Robert Amoanyi

**Affiliations:** a Department of Industrial Art, Ho Technical University, Ho, Ghana; b Department of Industrial Art, Kwame Nkrumah University of Science and Technology, Kumasi, Ghana

**Keywords:** Silica exposure, ceramic art studios, occupational health, artist well-being, risk perception

## Abstract

**Purpose:**

This study examines how Ghanaian ceramic artists and students perceive and respond to crystalline silica exposure in studio environments, situated within Ghana’s ceramic traditions where safety infrastructure and occupational health regulation remain uneven, and how these experiences influence health awareness and artistic identity in creative practice.

**Methodology:**

A qualitative interpretive phenomenological design was employed, using in-depth interviews with fifty Ghanaian ceramic artists and students with at least one year of active studio practice and exposure to clay-processing. Participants were selected regardless of respiratory symptoms to capture lived experiences. Participant observation was conducted in selected studios, and thematic analysis was applied to narratives to explore meaning-making and changing perceptions of silica-related health risks.

**Results:**

Findings identified a process through which artists integrate health awareness into their artistic identity, moving from initial disbelief about clay-related danger to increased bodily awareness, fear, and negotiating of normalized studio risk. Participants reported persistent chronic cough, chest tightness, and breathing difficulties associated with prolonged dust exposure, generatinganxiety and behavioral adaptations.

**Conclusions:**

Silica exposure is a cultural, educational, and institutional issue, requiring integrated health education, improved studio design, strengthened safety communication, and embedded occupational health practices to sustain long-term creative practice.

## Introduction

Pottery studios brim with creativity, community, and rich tradition, yet they also harbour an invisible threat. Among the most significant is exposure to respirable silica dust, a hazard embedded in the very materials used to produce ceramic art. Clay and glaze materials commonly contain silica (silicon dioxide, SiO₂), a mineral that becomes hazardous when airborne (Burgess, 1997). In everyday studio work, dry clay dust, glaze powders, and kiln emissions can release respirable silica particles (Sheth et al., 2026). Yet, within the creative rhythm of making, these invisible particles often go unnoticed, posing silent, cumulative risks that are rarely acknowledged in art education or studio culture. Inhaling these particles over time leads to silicosis, a progressive, incurable lung disease, as well as other respiratory illnesses and even cancer (Vanka et al., 2022; Weissman & Tallaksen, 2022). For example, pottery workers who handle silica flours and fire kilns (which convert clay silica to crystalline form) are **“identified as being at risk of crystalline silica exposure”** (Rana, 2023). Yet many artists feel safe amid clay dust. One Ghanaian potter confessed, *“I never thought clay could kill, it’s just earth under my hands,”* echoing a widespread disbelief about studio hazards.

Despite these well-documented hazards, silica exposure in artistic practice often remains overlooked. Within the creative rhythm of studio work, dust may be perceived as a normal or harmless by-product of making rather than a potential occupational hazard. This perception is reinforced by the culture of artistic training, where technical skills are frequently learned through apprenticeship or informal studio practice. In many cases, formal instruction in occupational health and safety is limited or absent in art education (Diamantis et al., 2026). As Hinkamp (2025) notes in public health reviews, arts skills are typically learned by apprenticeship with “no guarantee of substantial hazard prevention or even recognition,” and art schools lack health-safety requirements (Jain et al., 2018). Consequently, chronic health effectslike silicosis are largely “less well identified” in the arts sector (Williams, 2004).

While the biomedical risks of silica exposure are well established, less is known about how ceramic artists themselves perceive and interpret these risks within everyday studio practice. Understanding artists’ perceptions is important because risk awareness shapes safety behaviours, studio routines, and attitudes toward protective measures. Without insight into how artists make sense of occupational hazards within their creative environments, efforts to promote safer studio practices may remain ineffective or culturally disconnected from artistic practice.

In the Ghanaian context, ceramic training frequently occurs within informal apprenticeship systems, community-based pottery centres, and studio-led instruction, where structured occupational health modules are rarely formalised. Studies on Ghana’s informal artisan training sector suggest that skill transmission prioritises technique, production efficiency, and cultural continuity, while systematic safety instruction remains secondary or undocumented (Yeboah, 2024).

Although specific epidemiological data on silica exposure in Ghanaian ceramic studios are scarce, broader research on informal sector occupational health in Ghana indicates limited enforcement of safety standards (Osei, 2025), and low integration of preventive health education within craft-based learning environments. This structural gap suggests that silica-related risks may be similarly under-recognised within ceramic practice locally. Public health agencies have begun to recognise this gap: for instance, Washington State warns that toxic art materials can cause ailments, including silicosis (Kowalewski, 2022). But despite such alerts, awareness remains limited in studio culture.

This study therefore asks: How do ceramic artists perceive and navigate the health risks associated with silica exposure in their studio practice? To address this question, the study explores how ceramic artists and students perceive and make meaning of their exposure to silica dust within their studio environments. It investigates the lived experiences of health-related challenges connected to this exposure, particularly how these experiences shape their understanding of risk and well-being. Furthermore, the research examines the extent to which cultural norms, levels of awareness, and everyday studio practices influence the adoption or neglect of protective health measures. The study offers insight into how occupational hazards are internalised, managed, or overlooked in the context of artistic production. Building on this foundation, the enquiry also considers how broader contextual factors such as gender roles, age, and sociocultural expectations shape differing interpretations of risk and resilience. In doing so, the study illuminates how health awareness emerges within creative practice and informs more culturally grounded approaches to studio safety education, particularly within ceramic studio settings in Ghana.

## Literature review

Studies on silicosis and occupational silica exposure overwhelmingly focus on mining, construction, and large-scale industry. Hazards of crystalline silica are well-documented: inhalation causes lung fibrosis (silicosis) and is linked to tuberculosis, chronic bronchitis, and lung cancer (Sato et al., 2018; Subramaniyan et al., 2023). Despite this scientific clarity, awareness among ceramic artists remains uneven, with health risks frequently overshadowed by the aesthetic and expressive priorities of studio practice. Regulatory bodies (OSHA, NIOSH) set exposure limits, but many art and craft environments remain unregulated. Notably, a study of high-school ceramics classes measured respirable silica and found teachers frequently exceeded recommended limits (Fechser et al., 2014; Rossol, 2011). These findings underscore that even “non-industrial” arts settings can lead to hazardous exposure. Epidemiologic reviews identify pottery and ceramics among silica-risk occupations (Saeedizadeh et al., 2026). This classification underscores the need to view ceramic studios not only as creative spaces but also as occupational environments where long-term exposure demands informed safety practices and cultural shifts in risk perception. Epidemiologic evidence also suggests pottery workers have a higher risk of lung cancer, likely from dust exposure (Rapiejko et al., 2026). This troubling association invites deeper reflection on how artistic labour, often romanticised for its creativity, can obscure the very real and chronic health vulnerabilities embedded in everyday material engagement. In sum, quantitative studies affirm that ceramic materials and processes, mixing clay, dry trimming, glazing, and firing in kilns, can generate fine silica dust (Shojaee Barjoee & Rodionov, 2026).

Despite these facts, studies of creative workers often overlook health. Hinkamp (2025) argues that the arts, though employing millions, are *“poorly understood”* in health and safety research. He observes that chronic occupational illness in the arts is under-acknowledged. For example, many hazards in the visual arts (lead paints, solvents, silica) are similar to those in industrial settings, yet in art curriculum, they rarely require safety training (Wang, 2026). Such oversight points to a deeper systemic gap, where the celebration of artistic freedom often eclipses critical attention to the physical well-being of artists, forcing them to confront health hazards with minimal institutional support. This gap is mirrored in craft pedagogy: pottery courses focus on technique, not respiratory protection. In effect, silica exposure is an “invisible” hazard in studio fine dust that artists may normalise. Social studies of work hazards note that low visibility of risk often leads to denial or complacency until illness emerges (Demuth et al., 2022). Within creative environments, this normalisation becomes especially dangerous, as the intense sensory engagement with materials often masks hidden threats, turning health risks into an unnoticed yet persistent presence in artistic practice.

Risk perception theory holds that people evaluate hazards through experiential and cultural lenses. Workers often downplay familiar risks, especially when initial health effects are delayed (Marshall, 2020). In creative communities, a cultural script of dedication (“the show must go on”) can further minimise safety behaviours. This aligns with symbolic interactionism: studio norms frame silica dust as simply “part of working with clay,” shaping how artists collectively define risk. Meanwhile, occupational identity frameworks highlight how artisans may integrate self-sacrifice into their sense of craft. Monahan and Fisher’s concept of “sacrificial labour” describes how workers align personal sacrifice with career identity (Monahan & Fisher, 2020). In pottery, this may mean artists accept risks as inherent to their chosen trade. The review reveals a notable absence of qualitative studies examining how artists perceive silica risk, highlighting a significant gap. This study addresses that gap by listening to artists’ accounts of dust, health, and creativity.

## Theoretical framework

The analysis was grounded in theories of embodiment, risk perception, and identity. Embodiment theory (rooted in phenomenology) emphasises that bodily experience is fundamental to how individuals perceive the world (Fernandez, 2020). Merleau-Ponty argued that there is *“no hard separation between bodily conduct and intelligent conduct; rather, there is a unity of behaviour that expresses intentionality.”* (Godwins, 2024). In practical terms, an artist’s body is both subject and object: it both acts (shaping clay) and feels (sensing dust irritation). Silica exposure becomes meaningful through bodily symptoms (coughing, fatigue) and pre-reflective awareness of the air one breathes. The study also draws on risk perception theory, which recognises that subjective factors such as familiarity and control influence how dangers are understood. Artisans frequently interact with clay, making dust seem routine; theory predicts that such routine often lowers perceived risk unless a notable incident forces re-evaluation. Symbolic interactionism suggests that meanings of risk emerge through social context. In ceramics studios, shared language (e.g. “dust all over” vs. “silica hazard”) and norms will shape whether dust is seen as dangerous or benign. Finally, occupational identity theory informs how artists make sense of risks within their vocational self-concept. Many creative workers valorise struggle and sacrifice as part of their identity. Monahan and Fisher describe how workers may internalise *“sacrifice as the irreversible giving up of something else”* to achieve their career goals (Monahan & Fisher, 2020). These lenses were used to interpret how Ghanaian artists narrate their bodies’ experiences with dust, construct the meaning of danger, and negotiate safety as part of who they are.

## Methodology

**Figure 1.**
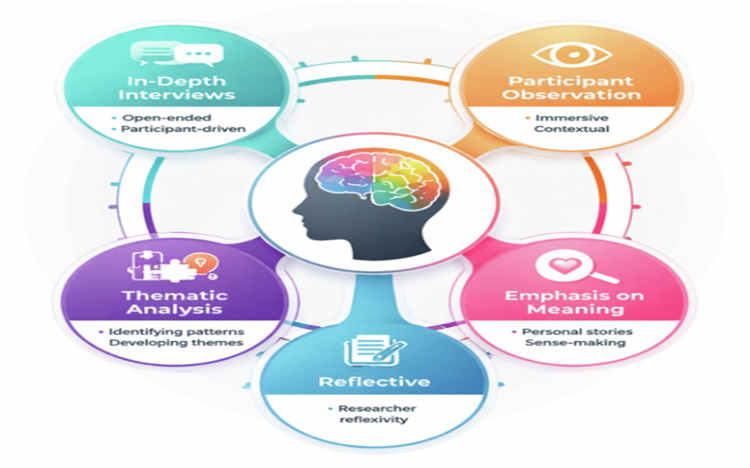
Interpretivist research design for exploring personal experiences. Source: Researchers Construct.

The study employed a qualitative, interpretivist design to explore personal experiences of silica exposure. This approach was appropriate because it seeks to understand how individuals make sense of their lived experiences within particular social and cultural contexts. Interpretive phenomenology acknowledges that meaning is co-constructed between researcher and participant. It enabled analysis to move beyond describing respiratory symptoms and studio routines to interpreting how silica exposure was normalised within studio culture and how health concerns became embedded in participants’ artistic identities. The framework, including data collection and analytical processes, is illustrated in Figure 1.

Participants were 50 individuals (aged 18–60) engaged in ceramic arts in Ghana, including professional ceramicists, studio technicians, and art students. Participants were purposively selected based on the following inclusion criteria: (1) minimum of one year of active ceramic studio practice; (2) regular involvement in dust-generating activities such as clay mixing, sanding, or kiln operations; and (3) willingness to share lived experiences related to studio health practices. Both individuals with and without self-reported respiratory symptoms were included to allow comparative insight. Those with less than one year of exposure or no active studio engagement were excluded. Recruitment was done from urban studios and craft workshops in Accra and Kumasi, ensuring a range of perspectives (gender, experience, studio size). The sample of 50 participants was selected to capture diversity across roles, settings, and experience levels. Data collection and analysis were conducted concurrently, with thematic saturation monitored through constant comparison. By the forty-second interview, no new themes emerged, and remaining interviews confirmed patterns across subgroups.

Data were collected through semi-structured interviews (lasting 45–90 minutes each) and non-participant observations. Interview topics probed artists’ studio routines, awareness of dust, health symptoms, safety practices, and emotional reactions. Studio observations (with field notes) documented ventilation, dust on surfaces, PPE use, and peer interactions around safety. In addition, the interview guide intentionally incorporated questions addressing demographic and sociocultural dimensions, including gender, age, and studio hierarchy. Participants were invited to reflect on whether these factors influenced their exposure patterns, perceived vulnerability, decision-making around protective measures, and willingness to challenge unsafe practices. Observational protocols similarly documented task distribution, leadership dynamics, and differences in safety behaviours across gender and age groups.

Interviews were audio-recorded and transcribed verbatim. In this study, a thematic analysis approach was used to interpret in-depth interviews and observational data, consistent with established qualitative methods in health research (Braun & Clarke, 2014). Two researchers independently coded an initial subset, compared and consolidated codes into a shared framework, and applied it to the full dataset, adding new codes as needed. Constant comparison was used as an iterative strategy within reflexive thematic analysis to refine codes and themes while staying grounded in participants’ lived, embodied, and occupational experiences. Codes were iteratively grouped into themes capturing bodily experience, risk perception, studio norms, and professional identity. Peer debriefing ensured biases were challenged and interpretations remained grounded, allowing descriptive codes (e.g. “dust irritation,” “mask resistance”) to develop into interpretive themes reflecting the normalisation of dust exposure and negotiation of health risks in ceramic studios.

Divergent or conflicting accounts, such as differences between veteran and early-career artists, were examined through constant comparison and treated as analytically significant variations. Rather than being reconciled into uniform patterns, these contrasts were retained to illuminate how experience level, studio hierarchy, and exposure history shaped differing constructions of risk, identity, and responsibility.

Throughout the research, the research team engaged in discussions to challenge biases and ensure credibility.

The research team includes trained ceramic art practitioners and art educators within the Ghanaian context. This positionality provided insider familiarity with studio culture, artistic identity, and apprenticeship systems, which facilitated rapport and contextual interpretation. However, it also carried potential biases, particularly the normalisation of dust exposure as “part of the craft” and assumptions regarding creative sacrifice. To manage these influences, the team engaged in reflexive memo-writing throughout data collection and analysis, explicitly interrogating how prior experiences in ceramic studios might shape interpretation. Regular peer debriefing sessions were conducted to question emerging themes, challenge taken-for-granted assumptions, and ensure that interpretations remained grounded in participants’ narratives rather than researcher expectations. To protect confidentiality, participants are referred to by pseudonyms (e.g. P5) and demographic descriptors.

## Reflexivity and researcher positionality

The research team comprises ceramic art practitioners and art educators working within the Ghanaian context. This positionality provided important advantages during the research process, particularly in facilitating rapport with participants and enabling a nuanced understanding of studio routines, apprenticeship systems, and the cultural meanings attached to ceramic practice. However, the researchers’ insider status also carried the potential risk of bias, especially the tendency to view dust exposure as an unavoidable aspect of ceramic work or to assume shared understandings of studio practices.

To address these concerns, the research team adopted a reflexive approach throughout the study. Reflexive memos were maintained during data collection and analysis to document assumptions, emerging interpretations, and potential researcher influences on the analytic process. In addition, regular peer debriefing sessions were conducted among the research team to critically examine coding decisions, challenge taken-for-granted assumptions, and ensure that interpretations remained grounded in participants’ narratives rather than researchers’ prior experiences. This reflexive process enhanced the transparency and credibility of the analysis by acknowledging how researcher positionality could shape the interpretation of participants’ experiences of silica exposure within ceramic studio culture.

Ethical approval was obtained from the Directorate of Research and Innovation (HTU- Ref No. HTU/DRI/EC2026-050), and all participants gave informed consent. Participants were assured that discussions of health would be treated sensitively; anyone expressing concern was provided with contacts for medical advice.

## Results

Several themes emerged from artists’ narratives about studio dust, health, and well-being. (see Table I for a summary of emergent themes and representative quotes).

**Table I.** Emergent themes from participant narratives on silica exposure in ceramic studios.

Theme	Description	Representative quotes
1. Initial unawareness and denial	Participants’ early lack of awareness about silica risks and tendency to dismiss potential hazards.	“Clay is like soil. How could soil kill you?” (P12) “I told myself it was just hard work, I’m not old enough to be sick.” (P30)
2. Bodily awareness and anxiety	Recognition of health symptoms over time (e.g. chronic cough, breathing issues) linked to studio exposure.	I would wake up coughing… it made me scared because I knew the dust was all over.” (P4)
3. Emotional tension and risk perception	Fear, hypervigilance, and emotional responses to understanding long-term hazards.	“I feel this scratch in my throat... my chest tightens. I get scared I’ll cough forever.” (P19)
4. Cultural and institutional normalisation	Studio culture, traditions, and art education practices that downplay or obscure silica risks.	“If my grandmum lived long, I suppose I will too.” (P33) “Nobody ever said, ‘You can get sick from that white stuff.’ It’s like an invisible enemy.” (P21)
5. Creativity-safety tension	Conflicts between creative practice and adherence to protective measures, such as PPE or cleaning routines.	“When I put on that mask and start throwing, I feel disconnected from the clay.” (P27)
6. Responsibility and safety engagement	Transition from ignorance to active adoption of safety practices and advocacy for protective behaviours in the studio.	“Now I brag that I always remind students to wash up. I feel more responsible as an artist.” (P9)“

### Study context and studio characteristics

The narratives presented in this study emerged from ceramic practitioners working across a range of studio environments in southern Ghana, including university teaching studios, independent artist studios, and small-scale community pottery workshops. The university studios were typically enclosed indoor spaces used for instructional activities such as throwing, glazing, and kiln firing, often accommodating multiple students simultaneously. Independent studios were smaller and artist-managed, with variable ventilation systems and cleaning practices. Community pottery workshops, often located in semi-open structures, relied heavily on traditional working methods and informal knowledge transmission.

Regionally, the studios were situated within humid tropical conditions typical of coastal and forest zones of Ghana, where high temperatures and limited mechanical ventilation may influence studio airflow and dust dispersion. These environmental and infrastructural conditions shaped everyday studio practices, including dry sweeping, sanding of greenware, and limited use of dust extraction systems. While the specific practices described reflect the Ghanaian studio context, many of the challenges identified such as limited safety training, normalisation of dust exposure, and tensions between creative practice and protective equipment are comparable to issues documented in ceramic studios internationally. Providing this contextual description enables readers to better evaluate how the findings may transfer to similar educational, artisanal, or small-scale ceramic production environments.

All participants initially expressed low expectations that clay could be harmful. One participant (P12, male studio potter) said simply, “Clay is like soil, how could soil kill you?” This reveals a deeply rooted perception that natural materials, especially those perceived as “organic” or familiar, are inherently harmless. From a risk perception perspective, this early disbelief reflects how familiarity and cultural proximity reduce perceived threat, even when an objective hazard exists. Clay, as a symbol of earth and creativity, was interpreted through socially constructed meanings rather than biomedical knowledge, illustrating how risk is culturally mediated rather than purely scientifically assessed. This interpretation aligns with what anthropologist Mary Douglas (1966) describes as the cultural construction of risk, where harm is not always evaluated by scientific criteria but through socially and symbolically informed beliefs.

This disbelief often shifted once participants began experiencing persistent physical symptoms most notably chronic cough, shortness of breath, chest tightness, and respiratory fatigue after prolonged studio exposure. Many reported noticing recurring morning cough and breathing discomfort after years of studio work. P4 (female pottery instructor) recalled, “I would wake up coughing in the morning, and it never went away. It made me scared because I knew the dust was all over.” This growing bodily awareness of dust inhalation brought anxiety. Interpreted through the lens of embodiment, these recurring symptoms functioned as lived, sensory knowledge. The body became the primary site through which risk was recognised and reinterpreted, transforming abstract information about silica into felt vulnerability. Another artist, P19, described what he termed “heart-whole fear”: “Every time I scoop dry clay or sand greenware, I feel this scratch in my throat. I tell myself, just breathe slowly, but my chest tightens. I get scared I’ll cough forever.” Here, the chronic cough and sensation of chest constriction were not isolated complaints but recurring embodied experiences reported across multiple interviews. Field observations corroborated these narratives. During routine studio cleaning sessions, visible plumes of fine dust were observed rising into the air when floors were dry-swept or when greenware was sanded without localised extraction systems. In several instances, windows were closed and mechanical ventilation was absent, allowing dust to linger visibly in the workspace. These conditions confirmed the persistent exposure described by participants.

**Figure 2.**
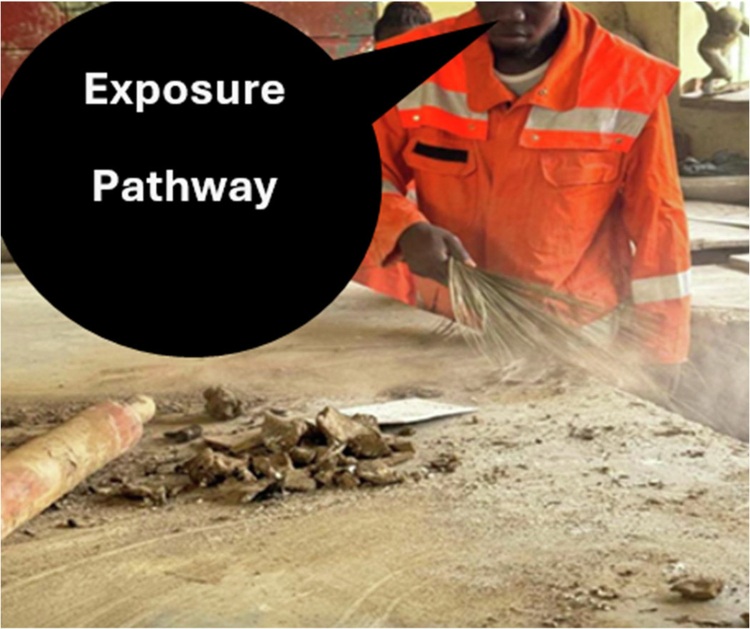
Illustration of silica dust exposure pathways during studio cleaning, showing inhalation through nasal and oral routes. Source: Authors’ field-based illustration, created for this study to protect participant anonymity.

The convergence of narrative accounts and observational evidence reinforces the embodied dimension of occupational risk, where environmental exposure is not only measured but physically experienced through breath, irritation, and fatigue. This embodied vulnerability is visually illustrated in Figure 2, which shows airborne clay dust being released during routine studio cleaning and the nasal and oral pathways through which it is inhaled.

In contrast, many participants noted that risk is often normalised in studio culture. Veteran artists said they grew up seeing white haze on shelves and clay under their feet every day. One elder potter (P33) said, “My mother and grandmother never had masks; they believed dust was just part of life. If my grandmum lived long, I suppose I will too.” Such family lore conveyed a sense that studio dust was “just how it is.” From a symbolic interactionist perspective, these inherited narratives operate as shared cultural scripts that define dust as ordinary rather than hazardous. Meaning is collectively produced and transmitted, shaping how artists interpret exposure within their occupational world. Students echoed this normalisation. One art student (P21) observed, “In school, they teach glazing and throwing, but nobody ever said, ‘you can get sick from that white stuff.’ It’s like an invisible enemy we don’t talk about.”

Observational data further revealed that safety signage was largely absent in the studios visited, and formal safety briefings were not conducted during practical sessions. Instructors typically emphasised technical skill acquisition over hazard awareness, reinforcing the institutional silence described by participants.

Importantly, the silence within formal art education (Theme 5) actively reinforced the cultural normalisation of risk (Theme 4). When institutions failed to provide explicit instruction about silica hazards, inherited studio beliefs remained unchallenged, allowing dust exposure to be interpreted as routine rather than preventable. Thus, cultural normalisation was not merely a family tradition but was institutionally reproduced through curricular omission.

This pattern further illustrates how risk perception is structurally shaped: when institutions fail to signal danger, the absence of warning becomes a powerful social cue that reinforces beliefs of safety. This cultural silence meant many only realised the danger through indirect cues such as medical consultations prompted by persistent cough or breathing discomfort, rather than through formal studio instruction. Participants also described a conflict between creativity and safety. Many found that protective measures felt at odds with their work. Wearing an N95 mask or respirator was often resisted. P27 (ceramics technician) explained, “When I put on that mask and start throwing, I feel disconnected from the clay”. Field observations confirmed that mask use was inconsistent across studio tasks. Masks were occasionally worn during glazing or wet clay mixing but were frequently neglected during high-dust activities such as sanding, dry sweeping, and studio cleaning, precisely when airborne dust concentration appeared highest. In some studios, masks were available but left hanging on walls or stored unused on shelves.

“I can’t smell it, I can’t feel it the same. It feels like a barrier to the art.”

Here, occupational identity becomes central. Protective equipment was interpreted not simply as a health intervention but as a disruption to the sensory and symbolic relationship between artist and material, revealing how professional self-concept influences safety behaviour. Some noted masks made breathing harder in Ghana’s warm climate, paradoxically intensifying awareness of their own respiration, especially among those already experiencing dust-related breathlessness.

Significantly, this creativity–safety tension (Theme 6) was intensified by the prior normalisation of risk (Theme 4) and the silence in art education (Theme 5). Because dust had long been framed as ‘part of the craft,’ protective equipment appeared excessive, disruptive, or even artistically inauthentic. In this way, the absence of formal safety discourse made precautionary practices seem culturally alien to studio identity, deepening resistance to PPE and wet-cleaning protocols.

Thus, what appeared as a personal discomfort with masks was also structurally shaped by entrenched studio norms and pedagogical gaps.

Emotionally, participants described a mix of anxiety, denial, and resilience. Anxiety often emerged after hearing about silicosis or witnessing colleagues develop lung conditions. P14 said, “When I heard a potter 10 years older than me had lung disease, I panicked. I found myself watching dust in the air, feeling my heartbeat in my throat.”

Yet others initially denied risk: “I told myself it was just hard work, I’m not old enough to be sick,” said P30. Denial (Theme 7) therefore functioned as both an emotional coping strategy and a cultural inheritance shaped by normalisation and educational silence. Gradually, many moved toward cautious respect for the hazard. Some forged humour or resilience. P8 now jokes about being “the mask lady,” meaning she habitually wears a respirator and reminds others to ventilate, even though she once ignored precautions.

This shift from obliviousness to vigilance often became part of artistic identity. P9 (a male potter) reflected: “I used to brag about never wearing protection; now I brag that I always remind students to wash up. I feel more responsible as an artist.”

This transition reflects a reconfiguration of occupational identity, where health-conscious behaviour becomes integrated into what it means to be a “responsible” artist. Through this process, embodiment reshapes identity, and risk awareness becomes part of professional self-definition. In this way, the lived experience of chronic cough, breathing difficulty, and chest discomfort transformed from a dismissed irritation into a catalyst for behavioural change and professional responsibility, integrating health consciousness into how artists understood themselves as craftsmen and mentors. Notably, the emergence of responsibility (Theme 8) represents a reversal of the earlier thematic chain: where silence and normalisation once shaped denial and tension, embodied illness experience disrupted that cycle and enabled new forms of safety-conscious artistic identity.

While core themes were shared across participants, variations emerged by professional status and gender. Veteran artists more often normalised dust exposure as part of occupational commitment, whereas students and early-career practitioners expressed greater anxiety and expectations of institutional safety guidance. Gender differences were subtler, with female participants more openly articulating precautionary awareness and some male participants emphasising resilience. These differences influenced how themes were framed rather than producing separate thematic categories.

## Discussion

The findings highlight how silica exposure in art studios is experienced through both body and culture. Initially, most artists did not recognise the hazard of clay dust, echoing what embodiment scholars describe as pre-reflective practice (Moya, 2014). Only when bodily symptoms (coughing, shortness of breath) appeared did they interpret dust as dangerous. This aligns with risk perception theory: invisible or familiar hazards (like studio dust) are often underestimated until a salient event triggers fear. The theme of *“I never thought clay could kill”* underscores this gap between everyday practice and latent risk.

The normalisation of dust in studio culture reflects symbolic interaction processes. Clay dust is given meaning through social narratives (“the older potters didn’t protect themselves, and they were fine”), which downplays danger. Such collective meanings delay precautionary action consistent with the literature on normalised deviance in workplaces. Participants’ accounts suggest an implicit cultural script: *creative labour involves sacrifice*. This resonates with Monahan and Fisher’s concept of sacrificial labour: yet our data reveal how this concept is reshaped within the Ghanaian context. Economic limitations, informal apprenticeship systems, and respect for senior artisans reinforce the notion that enduring discomfort is not merely personal choice but structurally and culturally embedded. Unlike industrialised contexts where sacrificial labour may be framed as individual dedication, in Ghanaian studios it intersects with resource constraints and institutional silence, highlighting the limitations of applying this concept universally.

Many participants described accepting discomfort or neglecting masks in the name of craft authenticity. In other words, an *occupational identity* formed around perseverance leads artists to tolerate hazards that others might avoid. Nevertheless, as the data show, this identity can evolve. Some artists reinterpret safety practices as part of their professional ethic. Wearing masks or improving ventilation became a source of pride or care for colleagues and students. A transformation is observed: initial *“body ignorance”* shifts to *“embodied awareness”*.

Participants showed divergent responses to silica risk: some normalised it through family or studio traditions, while others became hypervigilant after bodily symptoms. Embodied experiences, social norms, and hierarchical studio structures shaped whether individuals adopted protective practices. Those who successfully integrated PPE balanced creativity with safety, whereas others resisted due to entrenched norms, expectations of ‘sacrificial labour,’ and practical challenges, highlighting how body, culture, and context jointly influence risk perception and behaviour.

This study extends embodiment theory by showing how the body functions as both sensor and interpreter of risk within a culturally and structurally constrained environment. Recurring bodily symptoms such as coughing, chest tightness, and respiratory fatigue were not simply signs of exposure; they became the primary medium through which participants made sense of latent hazards. In other words, embodiment in this context is dialectical: the body experiences environmental risk while simultaneously shaping occupational identity and informing social learning about studio hazards. This insight adds to phenomenological understandings by demonstrating that bodily experience is inseparable from cultural norms, institutional practices, and professional self-conception.

However, this transition often came with emotional struggle: fear, guilt, and anxiety. Such emotional labour, worrying about future illness while trying to stay creative, reflects the psychological toll of sustained risk. It echoes findings in other creative fields where health issues (e.g. performance anxiety) are intertwined with career identity (Hinkamp, 2022). The participants’ stories illustrate how the need to *“keep making”* can conflict with the need to *“keep living safely.”*

Although few published studies specifically examine silica exposure within African or other low-resource artistic communities, this absence itself highlights a significant research gap. The structural features observed in Ghanaian studios' informal training pathways, limited occupational health enforcement, and culturally embedded normalisation of risk are consistent with patterns documented in informal craft sectors in other low-resource settings.

Finally, the silence around silicosis in art education intensifies the problem. As Hinkamp notes, without basic safety understanding, *“prevention and early recognition”* of chronic conditions is difficult. In Ghana, as in many countries, studio courses focus on skills, not hazards. Findings suggest that this gap must be addressed. Artists themselves became important teachers: several participants reported educating peers about dust after learning the hard way. This grassroots risk communication shows that workers can be agents of change in their communities, illustrating how occupational identity and social learning processes interact to mediate risk behaviour. However, participants’ accounts also revealed constraints that may limit such advocacy. Structurally, many studios operated without formal safety infrastructure, and economic limitations made investments in ventilation systems or high-quality respirators difficult. Culturally, hierarchical studio relationships and respect for senior artisans sometimes discouraged junior members from questioning established practices. In addition, deeply embedded narratives that dust is “part of the craft” could frame safety advocacy as unnecessary or overly cautious. These structural, economic, and cultural conditions show how symbolic interactionism and risk perception shape the negotiation of health risks, limiting the extent to which individual agency can transform occupational practices.

### Limitation of the study

This study has some limitations that should be considered when interpreting the findings. The research was conducted in urban ceramic studios in Accra and Kumasi, reflecting the institutional, cultural, and economic conditions specific to these settings. Studio environments in rural Ghana may operate under different apprenticeship structures, material constraints, or safety practices. Therefore, the findings should be interpreted within this localised context and not generalised to all ceramic studios in Ghana or other international contexts.

In addition, the study relied primarily on retrospective self-reported accounts of silica exposure and related health experiences, which may be influenced by recall bias or social desirability. The cross-sectional design, based on one-time interviews and observations, captures perceptions at a single point in time and cannot track changes in awareness or behaviour over time. Furthermore, the study did not include clinical health assessments or environmental exposure measurements to verify self-reported respiratory symptoms. Future research could incorporate longitudinal designs, environmental monitoring, and medical screening to provide a more comprehensive understanding of occupational health risks in ceramic studio environments.

#### Implications of the study

The findings of this study highlight important occupational health concerns related to silica exposure in ceramic studio environments. The participants’ experiences indicate limited awareness of dust-related risks, inconsistent use of protective equipment, and studio conditions that may facilitate dust accumulation. Based on these observations, several practical actions can be considered to improve safety in ceramic studio environments. While some recommendations are directly supported by participants’ accounts (such as the need for improved awareness and safer studio habits), others are proposed as broader preventive strategies informed by occupational health literature and best practices in studio safety. These suggestions should therefore be understood as potential directions for improving artist health and safety, rather than definitive policy prescriptions derived solely from the present data. The following are the proposed strategies for policy making

- **Short-Term Actions (Immediate and Low-Cost Interventions)**
  Artists can reduce silica exposure by consistently using appropriate PPE such as N95 or P100 respirators during dusty processes, adopting safer habits (e.g. avoiding dry sweeping, sealing dry materials, and cleaning clay residues from clothing), and implementing simple hygiene practices like wet mopping studio surfaces. Increasing awareness through peer workshops, studio posters, and safety briefings can further promote safer behaviours and encourage artists to view health protection as part of sustaining their creative careers.
- **Medium-Term Actions (Institutional Support)**
  Studios and educational institutions can improve safety by enhancing ventilation systems, including extractor fans or localised exhaust units, and integrating occupational health and safety education into art and ceramic training programs. Developing basic studio safety guidelines covering ventilation practices, PPE use, and wet-cleaning procedures can help standardise safer working conditions.
- **Long-Term Actions (Policy and Structural Interventions)**
  At a broader level, national occupational health guidelines for silica exposure in craft and creative industries may help raise awareness and promote safer studio environments. Institutional support, funding for ventilation and dust-control technologies, and artist health programs including counselling and peer-support initiatives could further strengthen long-term health and safety within the ceramic arts sector.

## Conclusion

Silica exposure in ceramic studios is often framed as a technical issue of dust control, yet this study demonstrates that it is equally a cultural, pedagogical, and experiential phenomenon. Through qualitative accounts from ceramic artists in Ghana, the research shows how occupational health risks become normalised within everyday studio practices, shaped by informal training systems, economic constraints, and shared narratives about dedication to craft. Participants’ experiences illustrate how awareness of risk frequently emerges through embodied symptoms rather than formal safety education, revealing the central role of the body as a site through which occupational hazards are recognised and interpreted.

Conceptually, this study contributes to qualitative health research by showing how embodied experience, occupational identity, and studio culture interact to shape risk perception and health behaviour in creative work environments. The findings extend discussions of occupational health beyond industrial settings, demonstrating how risk is negotiated in informal creative spaces where institutional regulation and safety training may be limited. By foregrounding artists’ lived experiences, the study highlights how cultural meanings attached to creative labour can simultaneously obscure and eventually reveal health risks.

These insights suggest that addressing silica exposure in ceramic practice requires more than technical interventions alone. Transforming studio environments will involve integrating health awareness into art education, encouraging collective responsibility for safety, and recognising artists as active interpreters of occupational risk. Listening to artists’ experiences is therefore critical for developing safety approaches that align with the realities of creative practice. In this way, the study contributes to broader conversations in qualitative health research about how occupational risks are culturally interpreted, embodied, and negotiated within everyday work environments.

## Data Availability

De-identified interview transcripts are not publicly available due to confidentiality agreements. Excerpts from transcripts have been included in this paper. Readers interested in the data may contact the corresponding author (cvicku@htu.edu.gh); access may be granted under strict confidentiality conditions.

## References

[cit0001] Braun, V., & Clarke, V. (2014). What can “thematic analysis” offer health and well-being researchers? *Journal of Qualitative Studies on Health and Well-Being*, *9*(1), 26152. 10.3402/qhw.v9.26152 PMC4201665 25326092

[cit0002] Burgess, G. L. (1997). Development and application of an exposure matrix for respirable crystalline silica in the British pottery industry: The University of Manchester (United Kingdom). 10.1016/s0003-4878(98)00005-2 9684560

[cit0003] Demuth, J. L., Vickery, J., Lazrus, H., Henderson, J., Morss, R. E., & Ash, K. D. (2022). Rethinking warning compliance and complacency by examining how people manage risk and vulnerability during real-world tornado threats. *Bulletin of the American Meteorological Society*, *103*(6), E1553–E1572. 10.1175/BAMS-D-21-0072.1

[cit0004] Diamantis, DV, Dalma, A, Balta, M, Karnaki, P, atsas K, K, Kandyliari, A, Pantazopoulou, A, Stefas, G, Kelemenis, P, Ikmpal, E, Sakowski, P, Dimitrova Atanasova, T, Veloudaki, A, & Linos, A (2026). Occupational Health and Safety Training: Perceptions of Barriers and Needs Among Municipal Urban Cleaners and Their Supervisors/Managers; A Qualitative Study Across 5 European Countries. New Solut. 10.1177/10482911261419468 41637221

[cit0005] Fechser, M., Alaves, V., Larson, R., & Sleeth, D. (2014). Evaluation of respirable crystalline silica in high school ceramics classrooms. *International Journal of Environmental Research and Public Health*, *11*(2), 1250–1260. 10.3390/ijerph110201250 24464235 PMC3945536

[cit0006] Fernandez, A. V. (2020). Embodiment and objectification in illness and health care: taking phenomenology from theory to practice. *Journal of Clinical Nursing*, *29*(21-22), 4403–4412. 10.1111/jocn.15431 32741016

[cit0007] Godwins, J. (2024). Philosophy of body: Merleau-Ponty and the philosophy of mind. *Twist*, *19*(1), 209–227.

[cit0008] Hinkamp, D. (2022). The hazards of work in the visual and performing arts. *Industrial Health*, *60*(5), 405–406. 10.2486/indhealth.60_500 35979580 PMC9539449

[cit0009] Hinkamp, D. (2025). Public health and the performing arts, *Perspectives in performing arts medicine practice ii: occupational health, public health, arts for healing* (pp. 3–14). Springer.

[cit0010] Jain, A., Leka, S., & Zwetsloot, G. I. (2018). Work, health, safety and well-being: Current state of the art. Managing health, safety and well-being: Ethics, responsibility and sustainability. 1–31. 10.1016/j.shaw.2017.05.001 PMC6111110 30363066

[cit0011] Kowalewski, K. (2022). *Prevalence rate of silicosis: A rising public health crisis*. University of Pittsburgh.

[cit0012] Marshall, T. M. (2020). Risk perception and safety culture: Tools for improving the implementation of disaster risk reduction strategies. *International Journal of Disaster Risk Reduction*, *47*, 101557. 10.1016/j.ijdrr.2020.101557

[cit0013] Monahan, T., & Fisher, J. A. (2020). Sacrificial labour: Social inequality, identity work, and the damaging pursuit of elusive futures. *Work, Employment and Society*, *34*(3), 441–456. 10.1177/0950017019885069 PMC7236897 32431474

[cit0014] Moya, P. (2014). *Habit and embodiment in Merleau-Ponty. In* (*Vol. 8*), pp. 542. Frontiers Media SA. 10.3389/fnhum.2014.00542 PMC4110438 25120448

[cit0015] Osei, P. (2025). *Human health risk assessment of artisanal engineering​,* *in Suame Industrial Area, Ghana*. Drexel University.

[cit0016] Rana, R. (2023). *Crystalline respirable silica exposures in a clay manufacturing company; An analysis of health risks and prevention strategies*. University of Wisconsin--Stout.

[cit0017] Rapiejko, A., Sosnowski, T. R., Sosnowski, K., & Jurkiewicz, D. (2026). Deposition of occupational aerosol particles in a three-dimensional adult nasal cavity model: An experimental study. Bioengineering, *13*(2), 132. 10.3390/bioengineering13020132 41749672 PMC12938197

[cit0018] Rossol, M. (2011). *The health & safety guide for film, TV & theater*. Skyhorse Publishing Inc.

[cit0019] Saeedizadeh, S., Assari, M. J., Ghorbani-Shahna, F., Faradmal, J., Karami, Z., & Mohraz, M. H. (2026). Probabilistic risk assessment of occupational exposure to respirable crystalline silica among ceramic workers in an industrial town in Iran: a Monte Carlo simulation approach. *Scientific Reports*, *16*, 6190. 10.1038/s41598-026-37121-w 41582202 PMC12905265

[cit0020] Sato, T., Shimosato, T., & Klinman, D. M. (2018). Silicosis and lung cancer: current perspectives. *Lung Cancer: Targets and Therapy*, 9, 91–101. 10.2147/LCTT.S156376 30498384 PMC6207090

[cit0021] Sheth, A., Kulkarni, N., Mansuri, M., & Viramgami, A. (2026). Exposure to Respirable Dust, Fine Particulates and Crystalline Silica and Comparative Respiratory Health Patterns Among Non-Smoking Workers in the Ceramic Industry.

[cit0022] Shojaee Barjoee, S., & Rodionov, V. (2026). Comprehensive risk profiling of occupational harmful factors in the ceramic industry: a case study from Iran. *Environmental Science and Pollution Research*, 33, 1–30. 10.1007/s11356-026-37443-2 41649671

[cit0023] Subramaniyan, V., Fuloria, S., Sekar, M., Shanmugavelu, S., Vijeepallam, K., Kumari, U., Fuloria, N. K. (2023). Introduction to lung disease, *Targeting Epigenetics in Inflammatory Lung Diseases* (pp. 1–16). Springer.

[cit0024] Vanka, K. S., Shukla, S., Gomez, H. M., James, C., Palanisami, T., Williams, K., Hansbro, P. M., Chambers, D. C., Britton, W. J., Ilic, D., & Horvat, J. C. (2022). Understanding the pathogenesis of occupational coal and silica dust-associated lung disease. *European Respiratory Review*, *31*(165), 210250. 10.1183/16000617.0250-2021 35831008 PMC9724915

[cit0025] Wang, L. (2026). Concept and characteristics of non-metallic mineral resources, *Manual of Mineral Material Science*. 442–448. Springer.

[cit0026] Weissman, D. N., & Tallaksen, R. J. (2022). *Silicosis​* *modern occupational diseases: Diagnosis, epidemiology, management and prevention* (pp. 58–73). Bentham Science Publishers.

[cit0027] Williams, H. (2004). Potted histories–cremation, ceramics and social memory in early Roman Britain. *Oxford Journal of Archaeology*, *23*(4), 417–427. 10.1111/j.1468-0092.2004.00219.x

[cit0028] Yeboah, C. (2024). *Artisanal skill acquisition and employment creation in the informal sector: A case study of Tema, Ghana*. University of Ghana.

